# Intergenerational patterns of digital use: Evidence from a large cross-sectional study

**DOI:** 10.1371/journal.pone.0353185

**Published:** 2026-07-08

**Authors:** Goran Erfani, Alison Steven, Lesley Young-Murphy, Wally Charlton, Holly De Luca, Gemma Wilson-MenzFeld

**Affiliations:** 1 Northumbria University, Faculty of Health and Wellbeing, School of Healthcare and Nursing Sciences, Newcastle-upon-Tyne, United Kingdom; 2 NHS North East and North Cumbria, Quadrant East, Cobalt Business Park, The Silverlink North, North Tyneside, United Kingdom; 3 Mental Health Services for Older People (MHSOP), Northumbria Healthcare NHS Foundation Trust, North Tyneside General Hospital, North Shields, United Kingdom; Universitas Prima Indonesia, INDONESIA

## Abstract

This study aims to determine the level of digital use across six generations (Greatest, Silent, Baby Boomers, and Generations X, Y and Z) within a district in North East England (population ~200,000). A cross-sectional descriptive study was conducted via an online and paper-based survey from February to May 2022, mailed to households in the district (N = 98,260). Respondents estimated their weekly use of digital tools and the internet at home. One-way ANOVA and Tukey-Kramer HSD tests were used to analyse differences in digital resource uses across six generations. To account for underlying sociodemographic characteristics, a follow-up ANCOVA analysis was also performed. Content Analysis was used for qualitative responses from an open-ended survey question. A total of 9,181 completed surveys were analysed. The sample was skewed towards older, homeowner adults (mean age 63, 60% female). Findings revealed that respondents spend less time online than other British cohorts. Baby Boomers and Generation X self-reported statistically significant differences in the level of digital use compared to all other generations. Younger generations (Y and Z) self-reported, on average, a larger amount of time spent on both digital tools and the internet. Members of Greatest and Silent Generations had the lowest hours spent on digital tools and the internet. The results suggest that public health initiatives should prioritise strategies bridging the digital divide between generations. Targeted training programs pairing younger, tech-savvy individuals with older adults could enhance digital literacy. Additionally, integrating user-friendly digital health platforms that cater to varying levels of technological proficiency will encourage wider adoption. These strategies not only foster intergenerational collaboration but also drive successful digital health and care transformations, ensuring equitable access to technological advancements for all age groups.

## 1. Introduction

The extent of digital utilisation varies across time and generations. In light of the COVID-19 pandemic, the reliance on digital tools such as smartphones and tablets has significantly increased. Different age groups employ these tools for varying purposes, including education, work-based activities, communication, and accessing public services from their homes [1–3]. Understanding these differences is crucial for decision-makers and policymakers in digital health and care transformation. Making assumptions that individuals across cohorts utilise digital tools in the same way could further exclude users from existing services, with the potential of widening the digital divide and further worsening health inequalities. This study responds to this need by examining generational patterns while acknowledging potential intra-generational variations in digital engagement. While older age is generally associated with lower digital engagement, it is important to recognise existing diversity *within* age groups, including potential high-usage individuals within older cohorts.

Theoretical understanding of the digital divide highlights three main factors impacting digital exclusion: access, skills and usage, and understanding the tangible outcomes of using technology that can result in offline benefits [4]. Both functional (access) and attitudinal (e.g., confidence and not recognising the benefits of technology) factors are predictors of technology use (or lack of) within older generations [5]. Studying patterns of digital usage across generations is crucial for better understanding how technology shapes society, informing targeted interventions to bridge digital divides, optimising products and services for diverse user groups, and ensuring effective policy development and regulation in the ever-evolving digital landscape.

The North East of England stands out as the region with the highest number of digitally excluded individuals in terms of access to the internet and usage patterns [6]. The UK evidence reveals that over half of the respondents in the North East (53%) are either ‘non-internet users’ or have ‘very limited internet usage’. This statistic highlights one of the most significant digital divides in the UK, separating extensive internet users from those who do not use it at all [6]. It is, therefore, essential to examine the digital usage patterns in this region and establish a robust evidence base to inform solutions for addressing digital exclusion locally and nationally. Understanding the challenges faced in the North East can provide valuable insights into digital use and barriers, with potential implications reaching far beyond regional confines.

The novelty of this study lies in its census-based approach to investigating digital use across all generations by targeting an entire district population. This strategy overcomes the limitations of previous research, which typically relied on convenience samples, online-only surveys, or examined restricted age groups. By offering multiple participation modes (paper, online, telephone) to every household and combining numerical data with qualitative responses, the study delivers a more holistic, outreach-oriented, and inclusive perspective that reflects the epistemological aim of capturing diverse digital experiences across all generational groups. This represents the largest UK-based study to date to use primary data to generate comprehensive, empirical evidence on generational differences in digital use, offering practical insights to help bridge the global digital divide in health and social care.

## 2. Background literature and theoretical framework

### 2.1. What is a generation?

A generation typically covers a range of 15–20 years. According to Generational Cohort theory [7], a ‘generation’ of individuals share similar political, economic, and social events during the early stages of life, and as a result develop a similar set of beliefs, values, and behaviours.

Within this study, the six generational cohorts have been defined based on suggestions made by [8,9]. Greatest Generation (born from 1901–1927), Silent Generation (born from 1928–1945), Baby Boomers (born from 1946–1964), Generation X (born from 1965–1980), Generation Y (born from 1981–1997), and Generation Z (born from 1998–2010). No one under the age of 18 years was included in this study.

Studying intergenerational differences in using digital resources is of paramount importance in today’s rapidly advancing technological landscape. Each generation has grown up in distinct time periods marked by unique societal, cultural, institutional, and technological influences [10,11]. Understanding how these generational cohorts engage with digital resources provides valuable insights into their needs, preferences, and behaviours for policymakers and practitioners. By comprehending generational differences, researchers and developers can focus on user-friendly human-technology interfaces, targeted communication and advocacy campaigns, and training approaches that resonate with specific age groups. Furthermore, healthcare institutions and providers can then tailor their strategies and offerings to effectively cater to diverse demographics. Analysing generational disparities in digital resource utilisation also fosters inclusivity, as it helps bridge the digital divide and ensures equitable access to technology across different age brackets.

### 2.2. Digital use and generational differences

It is important to consider generational factors within digital research for various reasons. Firstly, the introduction and adoption of technology occur generationally, over time. The way in which technology is used has evolved over time from organisational use in the workplace, to mobile smart technology that is used ubiquitously. For younger generations, technology has defined the way in which they communicate, socialise, create, and learn [12]. For others, there is, and has been, a steep learning curve which has ultimately changed the way in which they participate in communication, socialisation, creation, and learning. Differences between generations, or ‘digital natives’ and ’digital immigrants’ (those born prior to 1980), are argued to be as profound as structural brain changes which fundamentally impact the way in which individuals process digital information and learning [13,14]. However, others question the age-specific notion of this terminology through lack of clear-cut evidence of differences solely related to generation [12]. This study measures digital engagement primarily through self-reported hours of use (detailed in the next section), acknowledging that this may not fully reflect the nuanced, qualitative aspects of digital use highlighted in critiques of the ‘digital natives’ theory.

Secondly, age-related stereotypes and stigma are attached to digital technology use. Research has shown that stereotypes, particularly toward, and within, the older generation, impact real-world experiences of using technology. For example, a recent review [15] exploring the implementation and delivery of digital skills support programmes for older adults, highlighted the negative impact of internalised ageing stereotypes on an individual’s digital learning journey [16–19]. Conversely, there is the assumption that younger generations are digitally astute and digitally connected. However, while some evidence suggests the gap between adolescents’ digital access is lessening across socio-economic groups [20], other evidence illustrates that ‘data poverty’ remains prevalent through socioeconomic status, unstable housing, barriers to securing data contracts, and unstable employment [21]. This ultimately affects children and adolescents, as well as adults. As well as examining the importance of generational differences, it is also imperative that generational assumptions on digital access and use are questioned so as not to perpetuate them and further disadvantage individuals within these cohorts.

Whilst we know that older age is more likely to predict digital exclusion [22], it is not biographical age that is the predictor of digital use but contextual factors. A recent review by [23] evidences the importance of examining and considering inequalities and disadvantages through the life course as determinants of digital exclusion in later life. Within this review, a cohort study demonstrated digital exclusion as being related to decade group, rather than ‘structural’ (such as income, education, occupation) or ‘stage of life’ influences [24]. Rather than assuming that digital exclusion is a result of intrinsic ageing, it is important to consider generational factors when predicting or explaining digital exclusion [24].

As evident from the literature reviewed above, there is great importance in ongoing generational research into digital exclusion, and in recognising the ever-shifting generational understanding of digital use and digital needs. However, the existing underlying patterns of digital use across multiple generations are unclear and need further elucidation. Whilst existing evidence acknowledges the notion of generational digital differences, contradictions exist with research focussing on other sociodemographic factors which adds to the complexity of predicting and understanding digital use. Therefore, this study aimed to observe and analyse the existing level and patterns of digital use in North Tyneside (UK) across six generations (Greatest Generation, Silent Generation, Baby Boomers, Generations X, Y and Z). We hypothesise that there will be significant differences between different generations in using digital tools and the internet; specifically, younger generations will use more digital tools and the internet than older generations. Extrapolating from this hypothesis leads us to classify the existing intergenerational patterns of digital use according to each generation’s level of using digital tools and the internet.

## 3. Methods

A cross-sectional descriptive study was designed to survey intergenerational differences in the level of using digital resources across the district of North Tyneside in the North East of England, UK. Data were collected as a part of a larger mixed-method study investigating the scale and characteristics of digitally excluded residents and influential factors in predicting the experience of digital exclusion [22].

### 3.1. Data collection and participant recruitment

Data were collected between February and May 2022 from individuals living in North Tyneside who were at least 18 years old (the inclusion criteria). A package was mailed to all households (N = 98,260) within the district (census approach). The package included the invitation letter, the paper-based survey, the participant information sheet, and a prepaid envelope to return the completed survey. The research team also liaised with, and distributed surveys to, care homes and homeless shelters to increase the accessibility of this study. Furthermore, participants were able to complete this survey via telephone if preferred. Participants were informed about the purpose of the study via the information sheet and the confidentiality of their responses. A link to the online version of the survey (using the JISC platform) was also included in the package.

### 3.2. Ethical considerations

The study was granted approval by Integrated Research Application System (IRAS) for health and social care/community care research (no. 304555). The process of data collection, management, and analysis was conducted in accordance with the General Data Protection Regulation (2018) and the Personal Data Act (523/1999). Survey completion was voluntary, confidential, and anonymous. A participant information sheet detailing the study’s purpose, inclusion criteria, voluntary participation, data protection, confidentiality, withdrawal options, result dissemination, and anonymity was reviewed and approved by the Ethical Committee. This sheet was provided at the beginning of both the online and paper-based surveys. A written informed consent statement was presented on the first page of the survey, and participants were required to confirm their consent before proceeding to the survey questions.

### 3.3. Measurement

The data used in this study draws from a larger survey designed to assess digital exclusion across the study areas [22], which also included questions aimed specifically at measuring digital usage. The survey was developed using participatory co-design principles involving key stakeholders from health and social care sectors, academic experts, local authorities, charitable organisations and public groups. These stakeholders played an expert role in the design and pilot testing of the survey, as well as in recruitment and dissemination efforts. A panel of nine experts in the field (outside the research team) reviewed the survey prior to the survey dissemination to confirm its content validity for clarity and relevance. The survey was amended based on the suggestions and feedback. A pilot test with local residents (n = 35) was also conducted to double-check the format, style, and content of the survey before the data collection. The survey was refined and finalised after several reviews, input from the pilot-testing group, and discussions with the research team. A copy of the survey is available upon request. This collaborative approach not only enhanced the relevance and ethical standards of the research but also acknowledged the diverse expertise of stakeholders, ultimately leading to more impactful research outcomes.

The survey included three key sections. The first two sections focused on demographic and socioeconomic questions related to individuals and households, including information about education, employment, household income, and local primary care areas. Respondents were asked to provide their year of birth, which was then recoded into generational categories: 1901–1927 = Greatest Generation, 1928–1945 = Silent Generation, 1946–1964 = Baby Boomers, 1965–1980 = Generation X, 1981–1997 = Generation Y, 1998–2010 = Generation Z [8,9]. The last section included questions that collected self-reported time spent on digital resources. Respondents were asked to estimate independently the number of hours they spent using digital tools (including smartphones, tablets, laptops, desktop computers and all other digital devices) and, in a separate question, the internet at their homes during a typical week [25,26]. It is acknowledged that internet use typically occurs via digital tools; the two measures therefore capture overlapping but conceptually distinct dimensions of digital engagement (overall device use and specifically internet-based activity) rather than mutually exclusive behaviours. For those respondents who self-reported usage hours with one decimal point (fewer than 3% of total respondents), values were rounded to the nearest full number before mean calculations. While rounding may have slightly reduced precision, sensitivity checks confirmed that this had no meaningful impact on mean values or statistical significance. The final question on the survey included an optional open-ended question, asking participants if they wished to share any other comments about their experiences of using digital technologies. Participants were free to write as much, or as little, information as they wished.

### 3.4. Data analysis

Paper-based and online survey data were merged and integrated into a single master database. Then, the data was imported into SPSS version 28 [27] to perform all statistical analyses. The data’s homogeneity and normal distribution were assessed to determine their suitability for parametric statistical analysis techniques. Dependent variables (self-reported time spent on digital tools and the internet) were examined for multivariate outliers using Mahalanobis distance test and violations of normality and, if necessary, excluded from the statistical analysis [28]. The reason for removing outliers was to exclude implausible or inconsistent responses. Through manual inspection, we identified patterns indicative of data-entry errors (e.g., unrealistic reports of 200 hours per week for digital tools, which exceeds human capacity). We also conducted a sensitivity check and compared the key results with and without outliers. The findings remained consistent, indicating that removal did not distort observed generational patterns.

Descriptive statistics were first performed to describe the sample. Then, a one-way Analysis of Variance (ANOVA) was conducted to detect statistically significant differences in normally distributed average time spent on digital tools and the internet across six generation groups. The assumption of homogeneity of variance was tested using Levene’s test and when met (p > 0.05), Tukey-Kramer HSD post-hoc comparisons were used to identify statistically significant pairwise differences among generation means. Post hoc analysis was conducted separately for both dependent variables (use of digital tools and the internet). A non‑parametric Kruskal–Wallis test was also conducted as a robustness check to account for potential heterogeneity of variance across generational groups. The reported raw data for the dependent variables were used following outlier removal. Although some cohorts exhibited right-skewed distributions with SDs exceeding means (reflecting a floor effect among non-users), ANOVA is robust to such violations at large sample sizes (Tabachnick & Fidell, 2013), and the nonparametric Kruskal–Wallis results provide independent corroboration of the parametric findings. This floor effect is most pronounced in the Greatest Generation (n = 33), the oldest and smallest cohort, where it reflects a genuine concentration of non-users reporting zero hours rather than a data quality issue, with the remaining cohorts showing proportionally more balanced SD-to-mean ratios (see Table 2).

To examine whether the observed generational effects could be attributed to underlying differences in sociodemographic characteristics, a follow-up Analysis of Covariance (ANCOVA) was conducted. In this model, generation was entered as the independent variable, and time spent on digital tools and the internet was the dependent variable. Four potential confounding variables, i.e., education level (ordinal), employment status (categorical), household income level (ordinal), and residential area (categorical), were included as covariates.

Textual data were analysed using Content Analysis [29,30], focusing specifically on digital tools and internet use. All textual data were imported into NVivo 12 software to support analysis. For the purposes of the Content Analysis, all responses were divided into six generation groups. The researchers familiarised themselves with the data by reading through a few hundred free-text responses for each generation group. Initial search terms were developed using frequently discussed topics whilst carrying out this familiarisation task.

## 4. Results

### 4.1. Sample characteristics

A total of 9,181 surveys (7,513 paper-based and 1,668 online) were completed and returned to the research team, giving a response rate of 9.4%. However, only 8,372 of the responses were used in this study because the rest of the respondents did not meet the inclusion criteria or did not belong to any of the generations examined in this study. Outliers were defined as cases (n = 215) with Mahalanobis distance values exceeding the critical *χ*^2^ value of 14.02 at the 0.001 level. The final sample size was reduced to 8,372 after removing the outliers.

As Table 1 shows, most respondents were female (60%, n = 5038) and the average age was approximately 63 years (SD = 14.93; median = 66; min = 18; max = 101). According to the generational distribution, the average age was 98 years for Greatest Generation (n = 33); 82 for Silent Generation (n = 1443); 68 for Baby Boomers (n = 4358); 51 for Generation X (n = 1674); 34 for Generation Y (n = 829); and 22 for Generation Z (n = 35). The respondents were predominantly white (97%, n = 8147) with some level of education (89%, n = 7195) and mostly homeowners (88%, n = 7373) with an annual household income level of £40K or below (64%, n = 4564). The mean for household size was 2, varying from 1 to 8 persons in the household. Most respondents reported married, civil partnership, or co-habiting (62%, 5197). The rate of self-reported individual disability (or health conditions) and household disability (i.e., living with someone who has a disability) were respectively 36% (n = 2911) and 42% (n = 3437). Overall, our sample is, on average, a relatively old, mostly educated, predominantly white group of respondents who are homeowners with lower income levels, representing the main population characteristics of North Tyneside. Of the responses collected through outreach to care homes and homeless shelters, 30 were from residential care homes, 12 from sheltered accommodation, and 6 from hostels (n = 48 total). An additional 101 respondents did not disclose their accommodation tenure and may include further residents from non-standard living arrangements.

**Table 1:** Sample characteristics across the six generations.

Generations	Greatest	Silent	Boomers	X	Y	Z	Total
Sample size Female Age (Mean) White Educated Married Homeowner HH Income ≤ £40K INDIV Disability HH Disability	33 22 98 32 22 2 28 24 23 26	1443 746 82 1418 1047 682 1282 984 741 828	4358 2452 68 4266 3693 2758 3900 2749 1589 1890	1674 1182 51 1616 1586 1158 1464 541 415 513	829 607 34 781 814 589 682 246 134 171	35 29 22 34 33 8 17 20 9 9	8372 5038 63 8147 7195 5197 7373 4564 2911 3437

### 4.2. Generational differences in using digital tools and the internet

Based on the descriptive statistics, the overall sample means for the time spent on digital tools and the internet were 21.97 (SD = 18.70, median = 18; min = 0; max = 100) and 17.94 (SD = 17.69, median = 12; min = 0; max = 90) accordingly. The results of the ANOVA test revealed that a significantly higher level of using digital tools was detected among respondents of Generations X, Y and Z (see Table 2). Respondents of Generation Z reported the highest average score for time spent on digital tools (41.86 hours per week). The lowest average score for times spent on digital tools was reported among the respondents from Greatest Generation (5.79 hours per week). These variations in time spent on digital tools between the six generations were statistically significant (*F* = 566.31, p < 0.001).

**Table 2:** Inter-generational differences in the amount of time using digital tools and the internet across North Tyneside, UK (ANOVA).

Generations	N	Digital tools			Internet		
		Mean (SD)	95% CI	*F*	Mean (SD)	95% CI	*F*
Greatest	33	5.79 (10.13)	2.20–9.38	566.31*	3.94 (8.51)	0.92–6.96	495.45*
Silent	1443	10.19 (10.68)	9.64–10.74		7.97 (9.88)	7.46–8.47	
Boomers	4358	18.25 (14.66)	17.82–18.69		14.53 (13.61)	14.13–14.94	
X	1674	33.10 (20.47)	32.12–34.08		26.44 (20.43)	25.46–27.42	
Y	829	39.33 (21.13)	37.89–40.77		35.85 (20.86)	34.43–37.27	
Z	35	41.86 (18.69)	35.44–48.28		35.89 (20.39)	28.88–42.89	
Total	8,372	21.97 (18.70)	21.57–22.37		17.94 (17.69)	17.56–18.32	

Note: * indicates statistically significant results with p-value <0.001.

**Fig 1:**
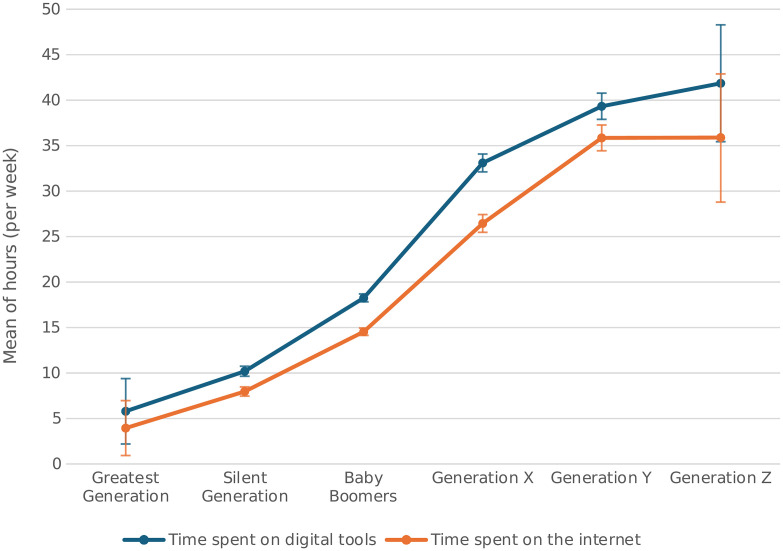
Mean plot for time spent on digital tools and the internet across six generations (with 95% CI).

The same pattern was observed when the six generations were compared for the time spent on the internet. As shown in Fig 1, the overall trend follows an S-curve from the oldest to the youngest generations, showing a progressive rise in internet use; however, the increase from Baby Boomers to Generation X is steeper than the rise between earlier cohorts, and Generations Y and Z exhibit very similar levels of internet use, creating an unexpected plateau at the younger end of the curve. The respondents belonging to Generations X, Y and Z reported significantly more time spent on the internet. Respondents of Generation Z reported the highest average score for time spent on the internet (35.89 hours per week), whilst the respondents from Greatest Generation revealed the lowest level (3.94 hours per week). Similarly, there were statistically significant differences in time spent on the internet between generations (*F* = 495.45, p < 0.001). Overall, the differences in the time spent on both digital tools and the internet for Generations X, Y and Z were higher and those of Greatest Generation lower than Silent Generation and Baby Boomers. Thus, younger generations were generally spending more time on digital resources than that of older generations. Table 2 lists the mean scores and inferential statistics across the six generations. As Table 2  shows, 95% confidence intervals were wider for the smallest cohorts (Greatest Generation and Generation Z), indicating reduced precision in these estimates, but the overall generational pattern remained unchanged.

The results of a non-parametric Kruskal–Wallis test also indicated a significant generational effect in time spent on digital tools (*H*(5)=2018.98, p < 0.001) and internet use (*H*(5)=1883.27, p < 0.001), with mean ranks increasing from the Greatest Generation to Generation Z (*see* S1 Table). These findings confirm that the observed differences are robust to violations of homogeneity-of-variance assumptions. Additionally, to further assess the potential for clustering within households, cases in which respondents shared the same postcode were cross-checked with reported household-level characteristics (e.g., household size, household tenure, etc). These checks showed that the number of respondents who could plausibly belong to the same household was very small.

The results of post hoc pairwise mean comparisons revealed a statistically significant variation in time spent on digital tools between Baby Boomers and all other five generations, i.e., Greatest, Silent, X, Y and Z (p < 0.001). Similarly, Generation X also reported a statistically significant mean difference from all other five generations (p < 0.05). The largest mean differences were between Baby Boomers and the younger cohorts of Generations Y and Z as well as Generation X versus the older cohorts of Greatest and Silent Generations. Only the variation between Silent and Greatest Generations, and Generations Y and Z were reported as statistically insignificant. In other words, the two oldest and youngest generations were found to have similar mean values for time spent on digital tools (see S2 Table).

A similar intergenerational pattern was also detected for time spent on the internet. A statistically significant variation in time spent on digital tools was detected between baby boomers and the other five generations, i.e., Greatest, Silent, X, Y and Z (p < 0.001). Respondents belonging to Generation X reported statistically significant differences from all other five generations (p < 0.01). Again, the largest mean differences were between Baby Boomers and the younger cohorts of Generations Y and Z as well as Generation X versus the older cohorts of Greatest and Silent Generations. Only the variation between Silent and Greatest Generations, and Generations Y and Z were reported as statistically insignificant. Specifically, the two oldest and youngest generations were found to be similar in terms of time spent on the internet (see S3 Table).

The ANCOVA results confirmed a statistically significant effect of generation on digital tool use (p < 0.001, partial η² = 0.047), even after controlling for covariates such as education, employment, income, and primary care location (see S4 Table). Among these covariates, employment status and household income also showed statistically significant associations with the time spent on digital tools, whereas education level had a minimal effect. Primary care location did not influence digital tool usage. The overall model accounted for approximately 32% of the variance in digital tool use.

Additionally, the ANCOVA revealed a statistically significant effect of generation (p < 0.001, partial η² = 0.062). This indicates that generational differences in internet use remained significant even after adjusting for the covariates (see S5 Table), confirming the existence of meaningful patterns in how different generations engage with the internet. Among the covariates, employment status and household income emerged as significant predictors of internet use, while education showed a more limited relationship. The primary care location examined did not appear to have a significant impact on internet usage patterns. This model explained over 26% of the variance in weekly internet use

### 4.3. Content Analysis of participants’ open-ended responses

Of the 9,181 participants, an impressive 2,016 (22% of participants) responded to the optional question (1,164 women, 849 men, and 3 other). The two themes generated in this analysis provide explanatory data that details reasons for the use of digital tools and the internet across generational cohorts (*see* Table 3). Whilst some of these reasons were similar across generations, others were more prominent in some generations than others.

**Table 3:** Themes generated from the data gathered in the open-ended survey question.

Theme	Sub-theme	Related search terms	Number of mentions
Digital access	Digital skills provision	Advice* Assist* Booklet Class Manual Pop-in Support* Teach Telephone	302
	Security and privacy	Cookies Fraud Hack Phishing Privacy Pop-ups Scam Security	103
	Health and disability	Accessibility Blind Deaf Disability Hearing Literacy People Person Reading Sight	44
Cost	Broadband provision	Afford* Cheap* Contract Cost* Expensive* Free Income* Upgrade*	523
	Digital devices	Afford* Cheap* Cost* Expensive* Income* Upgrade*	350

Note: * indicates search terms overlapped and captured data across multiple themes.

#### 4.3.1 Digital accessibility.

For many participants, the reasons for lower or non-existent use of digital tools and the internet were mainly due to a lack of digital access. This included the need for digital skills provision (302 mentions), concerns over security and privacy (103 mentions) and their personal experiences of health and disability (44 mentions). There were evident generational differences across digital accessibility issues.

Digital skills provision: Participants across Silent Generation, Baby Boomers, and a small sample of those within Generation X, expressed a strong interest in digital skills training or support to boost their proficiency with digital tools and the internet. Comments included:

*“Classes for the elderly” (Silent Generation)*

*“A manual with my iPhone. I feel I am not using its full capabilities” (Baby Boomers)*

*“Training courses would be good. I know they are available, but I don’t seem to have the time to attend them weekly. The digital world is overwhelming I’m slowly stepping into it” (Generation X)*

This contrasted greatly to Greatest Generation, and younger Generations of Y and Z. Across these generations, only one individual (within Generation Y) requested the need for digital skills provision.

*“I am not aware of all the things phones do, on-line/remote training for digital skills [would be useful] with an option to ask about personal gaps in my knowledge and questions” (Generation Y)*

Security and privacy: Although some individuals in Generations Y and Z did allude to concerns of hacking, online security, and particularly online privacy, was a concern predominantly for respondents in Silent Generation, Baby Boomers, and Generation X. Many of these concerns surrounded phishing, scams, and data security, as evidenced by comments such as:

*“Constant worry of cyber attacks and data protection.” (Generation X)*

*“I would sooner speak to people, not by technology. Everyone thinks that you are online/Facebook/E-mail/ plus all the scams that are about. This can lead to people being worried” (Silent Generation)*

Such fears impacted the ways in which individuals used digital tools and the internet, particularly financial and health-related applications.

*“I would NEVER use digital technology to access medical consultations. I consider it to be a dangerous practice which could easily lead to mis-diagnosis and I also have concerns about privacy issues” (Baby Boomers)*

Individuals specified conditions in which they would feel more secure online and would begin using more online services.

*“Assurance the data is private and securely stored” (Baby Boomers)*

Health and disability: Individual health conditions and disability impacted the amount of time spent on digital tools and the internet. Within this study, participants across Generations X, Y and Z did not reference their own health or disability as being a reason which impacted technology use. However, both Silent and Greatest Generations indicated that health and disability issues were prominent reasons for not using, or no longer using, digital technologies. Comments included:

*“Mum is deaf + blind so cannot use technology at all- even using a remote for her TV causes problems as the buttons are the small” (Greatest Generation)*

*“Partial sight limits use of digital technologies” (Greatest Generation)*

*“My main problem is I am partially sighted hence unaware as to whether I am pressing the correct keys, e.g., banking or paying anything online” (Silent Generation)*

#### 4.3.2. Cost.

Cost, particularly that of broadband provision (523 mentions) and digital devices (350 mentions), was an issue that impacted the use of digital tools and the internet for many. Unlike digital accessibility issues, these issues were experienced across generations.

Broadband provision: Individuals across all generations highlighted the prohibiting cost of broadband services, particularly that of higher speed. Comments reflecting this concern included:

*“Make broadband cheaper so it’s more accessible” (Greatest Generation)*

*“Cheaper broadband” (Baby Boomers)*

*“Reduce the prices for broadband and data” (Generation Z)*

High costs adversely affected digital connectivity. Not only this, but the reliability of broadband connections was also problematic and impacted digital use.

*“A more reliable broadband service” (Silent Generation)*

*“Faster broadband speeds. More reliable broadband connection also - usually cuts out at least twice every day for short periods” (Generation Y)*

Other individuals believed that broadband should be ‘free’ due to the necessity of being online, including free access to hotspots across North Tyneside.

*“Make [broadband] cheaper or provide hotspots for poor people or students to use” (Generation Y)*

*“More available, secure, free WiFi hotspots” (Baby Boomers)*

These issues were perpetuated by inadequate provider options and felt that switching between providers was difficult.

Digital devices: Similarly, the cost of digital devices was a concern across most generations in the sample (from the Silent generation to Generation Y). Often, the cost of devices was related to ‘devices breaking down’, or ‘becoming too old’. The cost of devices impacted how individuals were able to use technology as many were unable to upgrade their devices.

*“Big problem when devices break down. Cost of repairs etc” (Silent Generation)*

*“We aren’t able to get better quality items due to cost” (Generation Y)*

*“My devices are starting to be too old and apps etc are no longer working. The devices are fine in all other respects and the cost of updating are too much/not what I want to spend money on” (Generation X)*

#### 4.3.3. Emerging issues.

Although the content analysis did not reveal strong evidence of unexpected barriers such as digital fatigue or widespread intentional disconnection, a small but notable set of comments (e.g., “At my age (82), anything NEW stresses me out” and “Much prefer not to be digital – technologies when possible. Raises blood pressure, adds stress”) suggest that feelings of overload, stress, and even mental health concerns may exist among some older generations. While these comments were too infrequent to form a distinct theme, they point to emerging issues such as frustration, anxiety, and a desire to reduce reliance on technology that require further exploration, given their potential implications for well-being and social inclusion.

## 5. Discussion

### 5.1. Major findings

This is the largest regional UK-based study with respect to the sample size (n = 9,181) that explores intergenerational differences in using digital resources at a district level using primary data. As the findings of this study suggest, intergenerational differences in using digital tools and the internet exist between older and younger generations. Overall, our findings reveal high levels of digital use across Generations X, Y and Z, whereas the Greatest, Silent and Baby Boomers generations reported the lowest average levels. Much can be explained by the sense that the more generations become digitally native, the more their comfort level with, [11] and consumption of, [31,32] digital resources increase compared to previous generations. Several studies have linked the rapid adoption of digital resources, such as digital media, specifically since the 2000s, to the decline in the consumption of traditional legacy media, such as prints and television viewing [32–34]. From this perspective, younger generations of X, Y, and Z who tend to be more digitally savvy than older generations, spend more time online and less time with traditional media such as books, magazines, and TV. These results can also be understood through the lens of the fact that the internet offers greater benefits for improved employment opportunities and higher social status for younger generations [4]. This benefit may not necessarily correlate with the frequency of technology use but rather to the achievements frequent users gain in various important areas through their online engagement.

The qualitative findings also highlight differences in digital use across generations, namely, around the need for digital skills provision, health and disability, and privacy and security. Older generations, i.e., Silent, Greatest, and Baby Boomers (n = 4600, 78.8%), predominantly reported ‘online privacy and security’ as a potential barrier to online resource use. This was a huge concern for them and directly impacted the time spent on, and activities carried out, online. These results are consistent with studies indicating that age is the most significant predictor of online privacy or security concerns, and the older generations are more likely to be concerned about their online privacy [35,36]. Supporting older individuals to improve their online security systems, or knowledge, would benefit users by reducing their ‘risk perception’ and consequently increasing positive attitudes towards online resources [37]. Furthermore, according to the findings of the Content Analysis, many participants in this study suggested further access to educational/training solutions while less than 10% of respondents had attended a digital skills class in the last five years. Support must be accessible and affordable, and the reach/marketing of these services extends to those who are at higher risk of being digitally excluded.

The statistically insignificant results between the two youngest generations (Y and Z) and, similarly, between the two oldest generations (Greatest and Silent) indicate no fundamental differences in average time spent on digital resources within each pairing. It should be noted, however, that the non-significant results for the smallest groups (Generation Z, n = 35; Greatest Generation, n = 33) may reflect a Type II error due to limited statistical power rather than genuine behavioural equivalence, and should therefore be interpreted with caution. Nevertheless, these patterns are broadly consistent with wider literature suggesting that the ‘two modern digital generations’ of Y and Z [38] as well as the ‘two traditionalist generations’ of Greatest and Silent [39] ‘*are much more similar to each other than either are to the Baby Boom[ers]*’ [40] (p.924). Therefore, they are often regrouped and studied as a single generation, i.e., modernists and traditionalists, when their attitudes and behaviours towards digital resources are assessed. The qualitative findings of this study also indicated cost (including digital devices and data) as a consistent barrier impacting digital use across all generations.

The significant differences in digital usage between Baby Boomers and Generation X compared to all other generations can be linked to the socio-cultural contexts surrounding digital adoption [10,11]. The backgrounds and experiences of Baby Boomers and Generation X, referred to as ‘the forgotten generations’ [8], influence their values and needs related to work, privacy, and technology, often leading to distinct behaviours when compared to younger generations. Baby Boomers, characterised by their upbringing during economic prosperity and societal change, often prioritise values such as work ethic, privacy, and personal relationships. Their use of digital tools is often centred around maintaining connections with friends and family rather than immersive engagement in digital spaces. Generation X serves as a bridge between the analogue and digital worlds, utilising technology for efficiency while favouring traditional interactions about work-life balance and the importance of face-to-face interactions. In contrast, younger generations, including Generations Y and Z, often view digital engagement as a fundamental aspect of life, embracing social media and online interactions as integral to community building. They have grown up in an environment saturated with technology, where social media, instant communication, and online content consumption are commonplace.

Comparing our results with national data shows a clear difference in self-reported time spent on the internet by respondents in our North Tyneside sample compared to the wider population. On average, the respondents in our sample spend about 19 hours per week on the internet—approximately six hours less than the national average calculated by Statista Research Department [41] and five hours less than the national average measured by Ofcom [42] in September 2021. One explanation for these differences in time spent on the internet is that this study population included individuals clustered as ‘digitally excluded residents’; however, such groups are seldom represented, if at all, in existing studies. Targeting the population in North Tyneside by offering different modes of participation in our study (online survey, phone-based, and paper-based survey) enabled us to include and measure all ranges of internet users, including those not using the internet at all—again, something which is often omitted in research, which may tend to only focus on internet/digital device users. However, because our sample is older-skewed compared with national datasets (e.g., Statista; Ofcom), these comparisons require careful interpretation. Given the strong association between age and digital engagement, the lower usage observed in this sample could also partially reflect its age distribution rather than actual regional differences.

While self-reported time spent online provides a useful practical baseline indicator, it does not, by itself, equate to digital proficiency or skill [12]. Recent meta-analysis evidence shows that passive and active digital use generate different behavioural and well-being outcomes [43], highlighting why time-based metrics alone may obscure qualitative differences in digital engagement. For example, two older adults reporting similar online hours might have vastly different experiences: one passively browsing content, the other actively engaging telehealth services and learning platforms. Future research should explore these qualitative differences, as they matter considerably for policy decisions informed by our findings.

The 9.4% response rate may raise questions about non-response bias, particularly the potential under-representation of socio-economically disadvantaged groups at higher risk of digital exclusion, which may limit generalisability to the wider population. However, comparing our sample with North Tyneside’s demographic characteristics reveals reasonable alignment in several key areas, although our sample skews older than the North Tyneside population (median age ≈ 43; [44]), primarily because individuals under 18 were ineligible to participate in this study. In other respects, the sample reflects local demographics: most respondents identified as White (consistent with 95% of residents), over half were female, most were homeowners (mirroring the 63–64% owner-occupation rate), and the majority reported annual household incomes below £40K, reflecting the region’s low-to-mid income profile [44]. Additionally, the lower-than-average internet usage observed may reflect successful inclusion of digitally excluded individuals, who are rarely captured in online surveys, though it could also result from the sample’s older age profile, given the strong relationship between age and digital engagement [22,35]. Consequently, internet usage estimates and intergenerational comparisons should be interpreted as reflecting this adult sample rather than the entire district population.

While our findings broadly support generational cohort theory, which suggests that shared sociotechnical experiences shape digital behaviours [7,8], the observed patterns also question the rigidity of cohort boundaries. For example, similarities between Generations Y and Z, as well as between Greatest and Silent Generations, indicate that current generational classifications may oversimplify behavioural realities. Additionally, the distinct position of Baby Boomers, who differ significantly from both older and younger groups, complicates binary frameworks such as the “digital natives vs. digital immigrants” model [13,14]. This suggests that transitional cohorts may not fully align with these assumptions. Moreover, although digital divide theory emphasises disparities in access, skills, and outcomes [4], our qualitative responses reveal that cost-related barriers persist across all generations, indicating that structural constraints extend beyond age and are not fully addressed by existing models.

A discussion could emerge regarding which theoretical basis more effectively elucidates the observed patterns of intergenerational distinctions *between* (and within) the generations. The intergenerational differences identified in this study provide evidence-based support for the *learning theory of geragogy* [45,46], which supports the generational empowerment of older adults in learning and using digital tools/resources. For example, studies have highlighted the role of family-centred assistantship as a feasible solution to support the use (behavioural intention to use) of digital tools among older generations as well as improving intergenerational connectivity and relationships [47–49]. However, given the diversity present *within* each generational group (often referred to as intra-generational diversity), consistent with *critical geragogy theory* [15,50], it is also important to consider individual characteristics [51]. Understanding the differences *between* and *within* generations is essential in both formal and informal educational environments, especially as digital content-based applications become the norm in learning and teaching.

Generational differences in digital use can substantially influence the adoption and success of future digital health and social care transformation to achieve effective, desired outcomes [52]. If they are ignored or not addressed carefully, those differences can be a source of significant frustration and exclusion for older generations. Understanding each generational difference and considering the differences in healthcare policy-making and decision-making is crucial. Local authorities and decision-makers who acknowledge the differences and the priorities of each generation are likely to establish a digital environment that fosters inclusion, motivation, communication, and intergenerational interactions. It is critical to understand that failing to recognise and address intergenerational differences can contribute to social exclusion through increased digital inequalities, leading to socio-economic, and educational deprivations.

### 5.2. Implications for policy and practice

The results of this study offer some potential policy and practical implications regarding the value and use of existing intergenerational patterns in the level of digital use. Studying generational differences in using digital resources empowers us to adapt, innovate, and create a digitally inclusive society that meets the needs of individuals from all age groups. Intergenerational patterns should be considered for the transformation of existing and future digital health and social care services. Governments, healthcare organisations, educational institutions, and technology providers should ensure that healthcare services and healthcare education are accessible, effective, and sustainable across different generations of users. From this study we suggest:

*Accessibility and Inclusivity*—It is important to ensure that digital health and social care services are developed to be accessible to everyone, including individuals with disabilities (or health conditions) and older generations such as Baby Boomers, Silent and Greatest Generations who may have varying levels of digital literacy and comfort with technology. Digital services/products should provide options for different language preferences, adjustable font sizes, and easy navigation to accommodate a wide range of users. These recommendations align with our findings that older generations frequently reported accessibility challenges, particularly vision, hearing, and disability‑related barriers, affecting their ability to use digital tools. Additionally, our qualitative findings indicate that a small but notable subset of older adults experience feelings of stress, overload, or anxiety when engaging with digital tools, suggesting that inclusive service design must also consider psychological as well as functional barriers to digital engagement.

*Digital Skills and Education*—A priority should be implementing training programmes and user-friendly resources to improve digital skills among older generations, such as personalised ICT training interventions tailored for Baby Boomers and Silent Generation. This could be facilitated by promoting the use of digital health and social care tools for self-care and health management through educational campaigns with the assistance of the younger generation of family members. This reflects the substantial number of participants in the Silent, Baby Boomer, and Generation X cohorts who explicitly requested digital skills support.

*Affordability and Access*—It is important to ensure that the cost of digital services and devices is reasonable and accessible to all generations, considering the potential financial constraints faced by older generations. We suggest promoting reimbursement policies and insurance coverage for digital healthcare services. This recommendation is supported by our finding that cost, both broadband and device‑related, was reported as a barrier across all generations in our sample.

*Digital Participation and Sustainable Planning*—Considering the intergenerational differences in the level of digital use is important for ‘digital participatory planning’ [53]. Understanding and acknowledging generational differences in using and willingness to use digital resources contributes to better decision-making processes in disseminating and accessing information and how (and to what extent) different generations may digitally interact and participate in the planning processes. This is digital participation with a strong indication of how formal/informal processes in the North East of England can link different generations with planning processes: engagement, i.e., how different generations would be influenced by and influence digital resources and public services; and digital planning, including all programmes, methods, and initiatives that enable different generations to understand the new way of planning for our world which increasingly become digital.

### 5.3. Limitations and recommendations for future research

A limitation of this study is the lack of longitudinal data. Digital adoption is a dynamic process that evolves over time and varies across different contexts. A single cross-sectional snapshot may not adequately capture changes in behaviour over time. Conducting a follow-up study that tracks the same cohorts would significantly enhance the impact of the research. Our reliance on time-based measures (e.g., hours per week) provides a useful practical baseline, although it may not capture the qualitative nature of digital engagement. This approach is particularly relevant given critiques of the “digital natives” theory, which emphasise diversity *within* generations and the importance of skills and attitudes alongside usage time.

We also acknowledge that removing extreme digital usage as ‘outliers’ may exclude genuine behaviours within generations, especially among younger cohorts, rather than simply correcting data-entry errors. However, our sensitivity analyses showed that outlier removal did not affect observed generational patterns. Moreover, although evidence of household clustering appeared minimal (as discussed in 4.2 section), treating all responses as independent could still lead to underestimated standard errors in the presence of within-household similarity. Because the unit of analysis was the individual, household identifiers were not collected; therefore, we were unable to adjust for clustering in multilevel models. This is a structural limitation of this study’s design, as surveys were distributed at the household level, but responses were treated as independent individual observations.

Moreover, this study excluded an important demographic—Generation Alpha, which includes children and young people born after 2010. Studying Generation Alpha affords a unique opportunity to obtain a broader understanding of intergenerational patterns in digital usage across all surveyed generations. Collecting data on this cohort would permit an evidence-based review of current or planned digital inclusion strategies, addressing limitations and maximising their impact on educational outcomes, health outcomes, and future life chances of Generation Alpha. Future research should also investigate intergenerational digital interactions, such as family support in utilising technology. Additionally, employing a broader sample from multiple regions in the North East would provide a clearer understanding of digital usage, yielding findings that are more generalisable and comparable to the regional and national populations.

## 6. Conclusion

The present study demonstrated the existence of distinct disparities in digital resource usage among six generational groups. We make three notable contributions to this field of research. This is the largest study in the UK to gather primary data focussed on exploring intergenerational differences in the level of digital use. This study evidences generational differences in digital use across cohorts, with a high level of digital use across the three generations of X, Y, and Z, and the lowest digital use across Greatest and Silent generations. Baby Boomers and Generation X exhibited statistically significant differences in digital resource utilisation compared to all other generations. However, little differences existed between Generations X, Y and Z, and similarly Greatest and Silent Generations. These differences and similarities across generations are significant and impact policy decisions and practical applications when tackling digital exclusion. Moreover, our study highlighted that, overall, North Tyneside residents devoted less time to internet usage than other British cohorts.

These results underscore the importance of acknowledging intergenerational differences in digital resource usage when developing and providing online health/social care services and products, especially for older generations. Bridging the digital divide between generations is crucial as our society increasingly relies on digital platforms. The results suggest that public health initiatives should prioritise strategies that connect different generations by analysing and leveraging their patterns of digital tool usage. To enhance digital literacy across age groups, we suggest implementing targeted training programs that pair younger, tech-savvy individuals with older adults. Several comments explicitly requested help from family members, grandchildren, or someone to assist with digital tasks. For example, participants noted reliance on daughters or sons for online activities and expressed a desire for home visits or local support centres. These insights underscore the importance of intergenerational support as a practical and culturally acceptable solution to digital exclusion. Additionally, integrating user-friendly digital health platforms that accommodate various levels of technological proficiency will promote wider adoption. This strategy not only encourages collaboration among generations but also drives successful digital health and care transformations, ensuring that everyone can benefit from technological advancements.

The novelty of this study lies in applying a census approach to recruiting research participants—enabling the study to reach the entire population under study and giving everyone a chance to give their estimated level of using digital tools and the internet. This is an approach that can be taken internationally, particularly focusing on comparative analysis of generations across diverse nations. This will not only seek to understand differences between generations across the globe, but also differences within generations. In addition, the epistemic strategy of offering different modes of participation in this study (paper-based, online, or telephone) enabled the study to integrate individuals with different levels of digital skills and access to digital tools to participate in this study. This approach allowed us to be inclusive to a diverse group of digital users, including individuals who typically are ‘digitally excluded’ or not using the internet.

## Supporting information

S1 Table Mean ranks and Kruskal–Wallis test results for time spent on digital tools across generations. (DOCX)

S2 Table Tukey-Kramer HSD Post Hoc test results for multiple comparisons of different generations regarding time spent on digital tools. (DOCX)

S3 Table Tukey-Kramer HSD Post Hoc test results for multiple comparisons of different generations regarding time spent on the internet. (DOCX)

S4 Table ANCOVA Results – Tests of Between-Subjects Effects for time spent on digital tools (dependent variable). (DOCX)

S5 Table ANCOVA Results – Tests of Between-Subjects Effects for time spent on the Internet (dependent variable). (DOCX)
