# Supplementary material for: Intergenerational patterns of digital use: Evidence from a large cross-sectional study
Source: PLoS One. 2026 Jul 8;21(7):e0353185. doi: 10.1371/journal.pone.0353185 (PMC13345232; doi:10.1371/journal.pone.0353185)
Supplement: S1 Table — (DOCX) [file pone.0353185.s001.docx]

**Supporting Information**

S1 Table. Mean ranks and Kruskal–Wallis test results for time spent on digital tools across generations

| Generation | N | Mean Rank – Time spent on digital tools (per week) | Mean Rank – Time spent on Internet (per week) |
| --- | --- | --- | --- |
| Greatest | 33 | 1486.98 | 1376.08 |
| Silent | 1443 | 2469.14 | 2531.48 |
| Baby boomers | 4358 | 3842.75 | 3879.71 |
| Generation X | 1674 | 5571.16 | 5340.81 |
| Generation Y | 829 | 6193.54 | 6370.84 |
| Generation Z | 35 | 6573.21 | 6371.81 |
| Kruskal–Wallis H | — | 2018.98 | 1883.27 |
| df | — | 5 | 5 |
| p-value | — | **< .001** | **< .001** |

Note: Bold values indicate that the mean difference is statistically significant at 0.05 level or better.
