# Supplementary material for: Intergenerational patterns of digital use: Evidence from a large cross-sectional study
Source: PLoS One. 2026 Jul 8;21(7):e0353185. doi: 10.1371/journal.pone.0353185 (PMC13345232; doi:10.1371/journal.pone.0353185)
Supplement: S2 Table — (DOCX) [file pone.0353185.s002.docx]

**Supporting Information**

S2 Table. Tukey-Kramer HSD Post Hoc test results for multiple comparisons of different generations regarding time spent on digital tools

| **(I) Generation** | **(J)** Generation Comparison | **Mean Difference (I-J)** | **SD** | **p-value** |
| --- | --- | --- | --- | --- |
|  |  |  |  |  |
| Greatest | Silent | -4.403 | 2.846 | .634 |
|  | Baby boomers | -12.467^*^ | 2.825 | **<.001** |
|  | Generation X | -27.309^*^ | 2.842 | **.000** |
|  | Generation Y | -33.541^*^ | 2.870 | **.000** |
|  | Generation Z | -36.069^*^ | 3.922 | **.000** |
| Silent | Greatest | 4.403 | 2.846 | .634 |
|  | Baby boomers | -8.064^*^ | .491 | **.000** |
|  | Generation X | -22.907^*^ | .581 | **.000** |
|  | Generation Y | -29.139^*^ | .705 | **.000** |
|  | Generation Z | -31.667^*^ | 2.765 | **.000** |
| Baby boomers | Greatest | 12.467^*^ | 2.825 | **<.001** |
|  | Silent | 8.064^*^ | .491 | **.000** |
|  | Generation X | -14.843^*^ | .465 | **.000** |
|  | Generation Y | -21.075^*^ | .613 | **.000** |
|  | Generation Z | -23.603^*^ | 2.743 | **.000** |
| Generation X | Greatest | 27.309^*^ | 2.842 | **.000** |
|  | Silent Generation | 22.907^*^ | .581 | **.000** |
|  | Baby boomers | 14.843^*^ | .465 | **.000** |
|  | Generation Y | -6.232^*^ | .687 | **.000** |
|  | Generation Z | -8.760^*^ | 2.761 | **.019** |
| Generation Y | Greatest | 33.541^*^ | 2.870 | **.000** |
|  | Silent Generation | 29.139^*^ | .705 | **.000** |
|  | Baby boomers | 21.075^*^ | .613 | **.000** |
|  | Generation X | 6.232^*^ | .687 | **.000** |
|  | Generation Z | -2.528 | 2.790 | .945 |
| Generation Z | Greatest | 36.069^*^ | 3.922 | **.000** |
|  | Silent Generation | 31.667^*^ | 2.765 | **.000** |
|  | Baby boomers | 23.603^*^ | 2.743 | **.000** |
|  | Generation X | 8.760^*^ | 2.761 | **.019** |
|  | Generation Y | 2.528 | 2.790 | .945 |

Note: Bold values indicate that the mean difference is statistically significant at 0.05 level or better.
