# Supplementary material for: Intergenerational patterns of digital use: Evidence from a large cross-sectional study
Source: PLoS One. 2026 Jul 8;21(7):e0353185. doi: 10.1371/journal.pone.0353185 (PMC13345232; doi:10.1371/journal.pone.0353185)
Supplement: S3 Table — (DOCX) [file pone.0353185.s003.docx]

**Supporting Information**

S3 Table. Tukey-Kramer HSD Post Hoc test results for multiple comparisons of different generations regarding time spent on the internet

| **(I) Generation** | **(J)** Generation Comparison | **Mean Difference (I-J)** | **SD** | **p-value** |
| --- | --- | --- | --- | --- |
|  |  |  |  |  |
| Greatest | Silent Generation | -4.027 | 2.737 | .683 |
|  | Baby boomers | -10.594^*^ | 2.716 | **.001** |
|  | Generation X | -22.499^*^ | 2.733 | **.000** |
|  | Generation Y | -31.909^*^ | 2.759 | **.000** |
|  | Generation Z | -31.946^*^ | 3.772 | **.000** |
| Silent | Greatest | 4.027 | 2.737 | .683 |
|  | Baby boomers | -6.567^*^ | .472 | **.000** |
|  | Generation X | -18.472^*^ | .558 | **.000** |
|  | Generation Y | -27.882^*^ | .677 | **.000** |
|  | Generation Z | -27.920^*^ | 2.659 | **.000** |
| Baby boomers | Greatest | 10.594^*^ | 2.716 | **.001** |
|  | Silent Generation | 6.567^*^ | .472 | **.000** |
|  | Generation X | -11.905^*^ | .447 | **.000** |
|  | Generation Y | -21.315^*^ | .589 | **.000** |
|  | Generation Z | -21.352^*^ | 2.638 | **.000** |
| Generation X | Greatest | 22.499^*^ | 2.733 | **.000** |
|  | Silent Generation | 18.472^*^ | .558 | **.000** |
|  | Baby boomers | 11.905^*^ | .447 | **.000** |
|  | Generation Y | -9.410^*^ | .660 | **.000** |
|  | Generation Z | -9.447^*^ | 2.655 | **.005** |
| Generation Y | Greatest | 31.909^*^ | 2.759 | **.000** |
|  | Silent Generation | 27.882^*^ | .677 | **.000** |
|  | Baby boomers | 21.315^*^ | .589 | **.000** |
|  | Generation X | 9.410^*^ | .660 | **.000** |
|  | Generation Z | -.038 | 2.683 | 1.000 |
| Generation Z | Greatest | 31.946^*^ | 3.772 | **.000** |
|  | Silent Generation | 27.920^*^ | 2.659 | **.000** |
|  | Baby boomers | 21.352^*^ | 2.638 | **.000** |
|  | Generation X | 9.447^*^ | 2.655 | **.005** |
|  | Generation Y | .038 | 2.683 | 1.000 |

Note: Bold values indicate that the mean difference is statistically significant at 0.01 level or better.
