# Supplementary material for: Intergenerational patterns of digital use: Evidence from a large cross-sectional study
Source: PLoS One. 2026 Jul 8;21(7):e0353185. doi: 10.1371/journal.pone.0353185 (PMC13345232; doi:10.1371/journal.pone.0353185)
Supplement: S4 Table — (DOCX) [file pone.0353185.s004.docx]

**Supporting Information**

| S4 Table. ANCOVA Results - Tests of Between-Subjects Effects for time spent on digital tools (dependent variable) | | | | | | | | |
| --- | --- | --- | --- | --- | --- | --- | --- | --- |
|  | | | | | | | | |
| **Source** | **Type III Sum of Squares** | **df** | **Mean Square** | **F** | **Sig.** | **Partial Eta Squared** | **Noncent. Parameter** | **Observed Power^b^** |
| Corrected Model | 795738.812^a^ | 17 | 46808.165 | 190.126 | .000 | .322 | 3232.145 | 1.000 |
| Intercept | 304905.630 | 1 | 304905.630 | 1238.471 | <.001 | .154 | 1238.471 | 1.000 |
| Generations | 83027.105 | 5 | 16605.421 | 67.448 | <.001 | .047 | 337.241 | 1.000 |
| Education Level | 12454.493 | 4 | 3113.623 | 12.647 | <.001 | .007 | 50.588 | 1.000 |
| Employment | 54078.182 | 1 | 54078.182 | 219.656 | <.001 | .031 | 219.656 | 1.000 |
| Local Area | 963.906 | 3 | 321.302 | 1.305 | .271 | .001 | 3.915 | .351 |
| Household Income | 36709.113 | 4 | 9177.278 | 37.276 | <.001 | .021 | 149.106 | 1.000 |
| Error | 1674127.828 | 6800 | 246.195 |  |  |  |  |  |
| Total | 6158096.000 | 6818 |  |  |  |  |  |  |
| Corrected Total | 2469866.640 | 6817 |  |  |  |  |  |  |
| a. R Squared = .322 (Adjusted R Squared = .320) | | | | | | | | |
| b. Computed using alpha = .05 | | | | | | | | |
