# Supplementary material for: Intergenerational patterns of digital use: Evidence from a large cross-sectional study
Source: PLoS One. 2026 Jul 8;21(7):e0353185. doi: 10.1371/journal.pone.0353185 (PMC13345232; doi:10.1371/journal.pone.0353185)
Supplement: S5 Table — (DOCX) [file pone.0353185.s005.docx]

**Supporting Information**

| S5 Table. ANCOVA Results - Tests of Between-Subjects Effects for time spent on the Internet (dependent variable) | | | | | | | | |
| --- | --- | --- | --- | --- | --- | --- | --- | --- |
|  | | | | | | | | |
| **Source** | **Type III Sum of Squares** | **df** | **Mean Square** | **F** | **Sig.** | **Partial Eta Squared** | **Noncent. Parameter** | **Observed Power^b^** |
| Corrected Model | 593154.812^a^ | 17 | 34891.460 | 143.454 | .000 | .264 | 2438.712 | 1.000 |
| Intercept | 207447.551 | 1 | 207447.551 | 852.905 | <.001 | .111 | 852.905 | 1.000 |
| Generations | 109940.420 | 5 | 21988.084 | 90.402 | <.001 | .062 | 452.012 | 1.000 |
| Education Level | 5463.900 | 4 | 1365.975 | 5.616 | <.001 | .003 | 22.464 | .980 |
| Employment | 29341.105 | 1 | 29341.105 | 120.634 | <.001 | .017 | 120.634 | 1.000 |
| Local Area | 645.433 | 3 | 215.144 | .885 | .448 | .000 | 2.654 | .246 |
| Household Income | 16733.049 | 4 | 4183.262 | 17.199 | <.001 | .010 | 68.797 | 1.000 |
| Error | 1653927.531 | 6800 | 243.225 |  |  |  |  |  |
| Total | 4721203.000 | 6818 |  |  |  |  |  |  |
| Corrected Total | 2247082.343 | 6817 |  |  |  |  |  |  |
| a. R Squared = .264 (Adjusted R Squared = .262) | | | | | | | | |
| b. Computed using alpha = .05 | | | | | | | | |
